# C-reactive protein, renal function, and cardiovascular outcome in patients with symptomatic peripheral artery disease and preserved left ventricular systolic function

**DOI:** 10.3325/cmj.2015.56.351

**Published:** 2015-08

**Authors:** Mislav Vrsalović, Ksenija Vučur, Boris Car, Tomislav Krčmar, Ana Vrsalović Presečki

**Affiliations:** 1 School of Medicine, University of Zagreb, Zagreb, Croatia; 2 Division of Angiology, Department of Cardiology, Sestre Milosrdnice University Hospital Centre, Zagreb, Croatia; 3 Institute of Emergency Medicine of the Zagreb County, Zagreb, Croatia; 4 Faculty of Chemical Engineering and Technology, University of Zagreb, Zagreb, Croatia

## Abstract

**Aim:**

To investigate the prognostic role of C-reactive protein (CRP) and renal function for the occurrence of major adverse cardiovascular events (MACE) in patients with symptomatic peripheral artery disease (PAD) and preserved left ventricular ejection fraction (LVEF).

**Methods:**

The occurrence of MACE, defined as composite endpoint of acute myocardial infarction, urgent coronary revascularization, stroke, and death was assessed in 319 consecutive PAD patients admitted to the University Hospital between January 2010 and January 2014 (66.5% men, mean [±standard deviation] age 70 ± 10 years, mean ankle brachial index 0.58 ± 0.14) with normal LVEF (>50%). Multivariate Cox regression analysis adjusted for age, sex, traditional cardiovascular risk factors, anemia, polyvascular disease, critical limb ischemia (CLI), statin treatment, CRP (>5 mg/L), and impaired renal function (estimated glomerular filtration rate <60 mL/min) was applied to assess the independent predictors of MACE.

**Results:**

During median follow-up period of 24 months (interquartile range, 16-34 months), 77 patients (24%) experienced MACE. Compared to patients without MACE, these patients were older, more likely to have CLI, polyvascular disease, anemia, elevated CRP, and impaired renal function. In multivariate regression analysis, age (HR 1.04, 95% CI 1.01-1.07), polyvascular disease (HR 1.95, 95% CI 1.23-3.09), elevated CRP (HR 1.89, 95% CI 1.18-3.02), and impaired renal function (HR 1.68, 95% C 1.01-2.78) remained independent predictors of MACE. Patients with both impaired renal function and high CRP values on admission were 3.59 times more likely to experience MACE than patients with normal CRP and preserved renal function.

**Conclusion:**

Elevated admission CRP and renal impairment are independent predictors of MACE in symptomatic PAD patients with preserved LVEF.

C-reactive protein (CRP) is a simple marker, widely commercially available and regularly used in daily clinical practice. While CRP is well studied in acute coronary syndromes, its predictive role in symptomatic peripheral artery disease (PAD) is less clear (1). Conflicting data were published regarding the prognostic significance of admission CRP in patients with PAD (2-5). Its prognostic relevance may be affected by disease severity, duration of follow-up period, and inclusion of traditional risk factors.

Patients with chronic kidney disease have an increased risk of developing PAD (6,7). Data regarding the prognostic implication of impaired renal function in PAD patients are scarce. Despite interrelated pathophysiology of inflammation and renal function, their prognostic role has not been clarified in the clinical setting of symptomatic PAD. Left ventricular systolic dysfunction is associated with worse outcome and its prevalence is significantly higher in patients with peripheral vascular disease than in general population (8,9). However, this parameter was not included in prior outcome studies. Therefore, the aim of our research was to investigate the prognostic impact of CRP and impaired renal function for MACE in a cohort of consecutive patients with symptomatic PAD and preserved left ventricular systolic function.

## Patients and methods

Between January 2010 and January 2014 we studied 319 consecutive patients with symptomatic PAD (Fontaine stages IIB – 58%, III – 24%, and IV – 18%), admitted to the University Hospital. 16 patients were not included due to malignancy (12 patients) and concomitant autoimmune disorders (4 patients). The diagnosis of PAD was established by clinical examination, ankle brachial index measurement, duplex sonography and/or computed tomography or magnetic resonance, angiography, and confirmed with peripheral angiography using the criteria of the European Society of Cardiology and American College of Cardiology Foundation (10,11).

Baseline clinical characteristics and demographic data were recorded during the hospital stay and included general information (age, sex, weight, and height), data about cardiovascular risk factors, biochemical and hematological laboratory data, and data on comorbidities and medications. The diagnosis of hypertension was made in accordance with the European Society of Cardiology/European Society of Hypertension 2013 guidelines (12). Left ventricular ejection fraction (LVEF) was assessed using transthoracic echocardiography (Simpson’s method), and only patients with preserved LV systolic function (LVEF>50%) were included in the study. Cardiovascular disease (CVD), in addition to confirmed PAD, was defined as history of angina, myocardial infarction, coronary revascularization (percutaneous coronary intervention [PCI] or coronary artery bypass grafting [CABG]), history of stroke, transient ischemic attack, or carotid revascularization.

High sensitivity CRP was determined on admission by immunoturbidimetric method (Olympus, Dublin, Ireland). Estimated glomerular filtration rate (eGFR) was calculated using the Modification of Diet in Renal Disease formula. Baseline anemia was defined according to World Health Organization criteria (hemoglobin level <13 g/dL for men and <12 g/dL for women) (13).The investigation was performed in accordance with the Declaration of Helsinki and was approved by the University Hospital Ethics Committee.

Patients were followed up for the occurrence of the first major adverse cardiovascular event (MACE), defined as composite endpoint of acute myocardial infarction, urgent revascularization (PCI or CABG), stroke, and death. Peripheral vascular events (revascularization procedures or amputations) during follow-up were not considered as a study endpoint. The median follow-up was 24 months (interquartile range, 16-34 months). Patients were followed up in the outpatient clinic at 3, 6, and 12 months after discharge and then annually. Furthermore, periodic telephone interviews were performed to determine the occurrence of events. Mortality was documented by death certificates or by reviewing the hospital records. Outcome was assessed by independent observers, who were blinded to patients’ laboratory and clinical data.

Continuous normally distributed variables are expressed as mean (±standard deviation) and not-normally distributed variables as medians (interquartile range). Differences between the groups were analyzed with *t* test and Mann-Whitney test for continuous variables and with χ^2^-square test for categorical variables. Normality of distribution was tested with Kolmogorov-Smirnov test. The ability of CRP and eGFR to predict MACE was tested by receiver-operating characteristic (ROC) analyses. Kaplan-Meier analysis with log-rank test was performed and comparison was based on optimal cut-off points of CRP (dichotomized to > or<5 mg/L) and eGFR (dichotomized to > or<60 mL/min) based on ROC analyses and previously published data (2). Cox proportional-hazards regression analysis was performed to determine the independent predictors of MACE and results were expressed as hazard ratios (HR) and 95% confidence intervals (CI). Covariate selection included known correlates of poor cardiovascular outcome and those that were found to be significant in the univariate analysis (univariate *P* value <0.100), namely age, sex, traditional cardiovascular risk factors, anemia, history of cardiovascular disease, critical limb ischemia, and statin treatment. CRP and eGFR were entered in multivariate analysis as binary variables. The value of *P* < 0.050 was considered statistically significant. Statistical analysis was performed using MedCalc® Version 11.3.1.0 (MedCalc, Ostend, Belgium).

## Results

The baseline characteristics of the 319 symptomatic PAD patients are summarized in Table 1. During median follow-up period of 24 months (interquartile range, 16-34 months), 77 patients (24%) had a MACE, with 20 myocardial infarctions, 8 percutaneous coronary interventions/CABG procedures, 11 strokes, and 38 deaths. In the univariable analysis older age (*P* < 0.001), critical limb ischemia (*P* = 0.012), anemia (*P* = 0.018), history of cardiovascular disease (*P* = 0.002), decreased eGFR (*P* < 0.001), and higher CRP values (*P* = 0.009) were significantly associated with MACE (Table 2).

**Table 1:** Baseline characteristics of 319 patients with symptomatic peripheral artery disease

Characteristic	
Age (years)*	71 (63-78)
Male sex, n (%)	212 (66.5)
Ankle brachial index^†^	0.58 ± 0.14
Systolic blood pressure (mmHg)*	140 (130-154)
Diastolic blood pressure (mmHg)*	80 (80-90)
Heart rate (beat/min)*	75 (67-80)
Body mass index (kg/m^2^)*	27 (25-30)
Hypertension, n (%)	277 (87)
Diabetes mellitus, n (%)	172 (54)
Smoking, n (%)	173 (54)
Dyslipidemia, n (%)	242 (76)
Polyvascular disease, n (%)	132 (41)
Critical limb ischemia, n (%)	134 (42)
Anemia, n (%)	67 (21)
High sensitivity C-reactive protein (mg/L)*	4.5 (2.2-10.0)
Estimated glomerular filtration rate (mL/min)^†^	63.4 ± 18.3
Left ventricular ejection fraction (%)^†^	57.0 ± 5.5
Statin therapy, n (%)	194 (61)
Antiplatelet therapy, n (%)	299 (94)

*Median (interquartile range).

†Mean ± standard deviation.

**Table 2:** Univariate and multivariate Cox proportional-hazards regression analysis for major adverse cardiovascular events*

	Univariate		Multivariate	
Variable	HR (95% CI)	*P*	HR (95% CI)	*P*
Age	1.048 (1.022-1.075)	<0.001	1.036 (1.009-1.065)	0.010
Female sex	1.254 (0.795-1.979)	0.333	-	
Hypertension	2.905 (1.067-7.910)	0.038	-	
Diabetes mellitus	1.084 (0.690-1.703)	0.727	-	
Smoking	1.124 (0.715-1.765)	0.615	-	
Dyslipidemia	1.077 (0.633-1.831)	0.786	-	
Critical limb ischemia	1.798 (1.138-2.840)	0.012	-	
Coronary artery disease	1.499 (0.947-2.375)	0.085	-	
Polyvascular disease	2.045 (1.306-3.201)	0.002	1.945 (1.225-3.088)	0.005
hsCRP (>5 mg/L)	1.861 (1.165-2.972)	0.009	1.887 (1.179-3.022)	0.008
eGFR (<60 mL/min)	2.464 (1.551-3.912)	<0.001	1.677 (1.012-2.778)	0.045
Anemia	1.775 (1.103-2.855)	0.018	-	
Statin therapy	1.001 (0.635-1.578)	0.996	-	

*HR – hazard ratio; CI – confidence interval; hsCRP – high sensitivity C-reactive protein; eGFR – estimated glomerular filtration rate.

**Figure 1:**
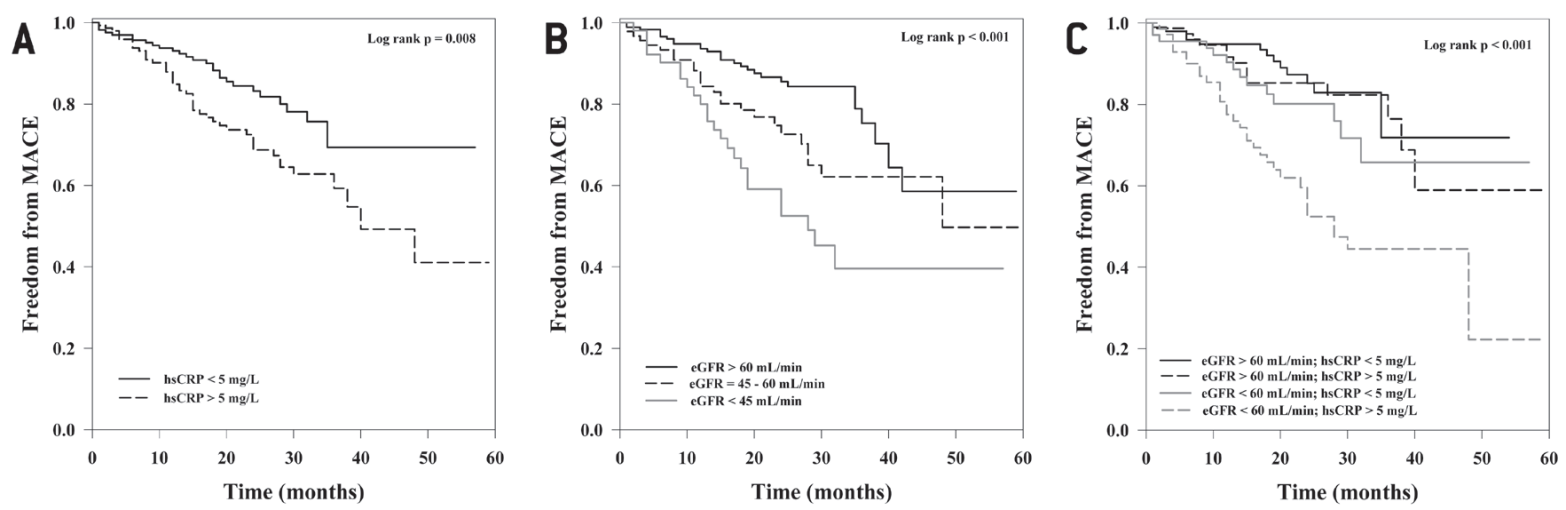
Cumulative major adverse cardiovascular events (MACE) free survival according to: (**A**) baseline high sensitivity C-reactive protein (hsCRP), (**B**) estimated glomerular filtration rate (eGFR), and (**C**) combined effect of baseline hsCRP and eGFR in 319 patients with symptomatic peripheral artery disease.

CRP (*P* = 0.008) and eGFR (*P* < 0.001) predicted freedom from MACE (Figure 1A and 1B). In logistic regression analysis, critical limb ischemia (odds ratio [OR] 4.59, 95% CI 2.76-7.63; *P* < 0.001), anemia (OR 2.15, 95% CI 1.14-4.05; *P* = 0.017), and hypertension (OR 3.44, 95% CI 1.48-7.97; *P* = 0.004) were significantly associated with elevated CRP values, while age (OR 1.09, 95% CI 1.07-1.13; *P* < 0.001) was associated with impaired renal function. Baseline hemoglobin levels negatively correlated (Spearman correlation test) with CRP values (ρ = -0.232; *P* < 0.001), and positively with eGFR (ρ = 0.328; *P* < 0.001).

In multivariable Cox regression analysis, age, polyvascular disease, elevated CRP, and impaired renal function independently predicted MACE (Table 2). Patients having both impaired renal function and high CRP values at baseline were 3.59 times (95% CI = 1.89-6.83; *P* < 0.001) more likely to experience MACE than patients with normal CRP and preserved renal function (Figure 1C).

## Discussion

This study found that the baseline CRP, renal function, age and polyvascular disease were independent predictors of MACE. Additionally, patients with both renal impairment and elevated CRP values at baseline had the highest risk for MACE.

PAD is one of the most frequent manifestations of atherosclerosis and is associated with a very high risk of ischemic events, ie, myocardial infarction, stroke, and death (14). Therefore, besides early diagnosis and treatment it is important to timely assess the risk for cardiovascular events.

While CRP is well studied in acute coronary and acute aortic syndromes (1,15,16), its prognostic role in symptomatic PAD is still controversial (5). In line with our results, Owens et al showed independent prognostic value of CRP (>5 mg/L) for all-cause mortality in PAD patients undergoing bypass surgery (2). Schlager et al showed that baseline CRP (>8.8 mg/L) predicted MACE in patients who underwent angioplasty due to symptomatic PAD (3). On the other hand, Breveti et al showed that CRP did not predict myocardial infarction or stroke (4). Of note, left ventricular systolic function, although associated with poor outcome was not investigated in these studies. We investigated patients with preserved ejection fraction who received conservative or endovascular treatment together with patients who underwent lower extremity bypass operation and evaluated a broad spectrum of adverse cardiovascular outcomes which makes our sample similar to the population treated in the daily clinical practice.

Chronic kidney disease (CKD) is associated with adverse outcomes in patients with cardiovascular disease, namely acute coronary syndromes, after percutaneous coronary intervention and coronary artery bypass surgery (17,18). However, little attention has been paid to the prognostic role of renal function in symptomatic PAD patients, keeping in mind that CKD and PAD share some common risk factors (6). Renal impairment was found to predict all-cause mortality in patients with symptomatic PAD (19). We believe this is the first report that showed the independent prognostic role (defined as the occurrence of MACE) of impaired renal function in patients with severe PAD, regardless of conventional risk factors, disease severity, and inflammation.

Patients with polyvascular disease have an increased risk of death (20). The results from the REduction of Atherothrombosis for Continued Health registry showed that patients with atherosclerotic disease affecting two or more vascular beds had significantly worse prognosis than patients with single arterial bed disease (21). In our study, patients with polyvascular disease were two times more likely to suffer adverse outcomes than those with isolated peripheral artery disease.

A limitation of our study is that we did not routinely check parathyroid hormone levels in PAD patients, which might be a contributing factor to the worsening of atherosclerotic process. In conclusion, our data show that elevated admission CRP values and renal impairment are independent prognostic factors of adverse clinical outcome in patients with symptomatic PAD and preserved LVEF. The coexistence of impaired renal function and inflammation substantially increase the risk of future ischemic events.
